# Antagonistic Activity of *Lactobacillus* Isolates against *Salmonella typhi In Vitro*

**DOI:** 10.1155/2013/680605

**Published:** 2013-09-29

**Authors:** Amira Abdel-Daim, Nadia Hassouna, Mohamed Hafez, Mohamed Seif Aldeen Ashor, Mohammad M. Aboulwafa

**Affiliations:** ^1^Department of Microbiology & Immunology, Faculty of Pharmacy, Modern Sciences and Arts University, Cairo 12611, Egypt; ^2^Department of Microbiology & Immunology, Faculty of Pharmacy, Ain Shams University, Al Khalifa Al Maamoun Street, Abbassia, Cairo 11566, Egypt; ^3^Department of Pharmaceutical Microbiology, College of Pharmacy, Taif University, Saudi Arabia

## Abstract

*Background*. Enteric fever is a global health problem, and rapidly developing resistance to various drugs makes the situation more alarming. The potential use of *Lactobacillus* to control typhoid fever represents a promising approach, as it may exert protective actions through various mechanisms. *Methods*. In this study, the probiotic potential and antagonistic activities of 32 *Lactobacillus* isolates against *Salmonella typhi* were evaluated. The antimicrobial activity of cell free supernatants of *Lactobacillus* isolates, interference of *Lactobacillus* isolates with the *Salmonella* adherence and invasion, cytoprotective effect of *Lactobacillus* isolates, and possibility of concurrent use of tested *Lactobacillus* isolates and antibiotics were evaluated by testing their susceptibilities to antimicrobial agents, and their oxygen tolerance was also examined. *Results*. The results revealed that twelve *Lactobacillus* isolates could protect against *Salmonella typhi* infection through interference with both its growth and its virulence properties, such as adherence, invasion, and cytotoxicity. These *Lactobacillus* isolates exhibited MIC values for ciprofloxacin higher than those of *Salmonella typhi* and oxygen tolerance and were identified as *Lactobacillus plantarum*. *Conclusion*. The tested *Lactobacillus plantarum* isolates can be introduced as potential novel candidates that have to be subjected for *in vivo* and application studies for treatment and control of typhoid fever.

## 1. Background

Typhoid fever continues to be a public health problem in developing countries where infections are endemic, since it has been an important cause of illness and death, and it has been exacerbated by the emergence of antibiotic resistance. Although chloramphenicol has been the “gold standard” of therapy, a widespread plasmid-mediated resistance emerged in *S. typhi*, with outbreaks in 1970 [1]. This led to the replacement of chloramphenicol by ciprofloxacin as the drug of choice. However, *Salmonella typhi *and *paratyphi A *acquired resistance to fluoroquinolones and other antimicrobial agents, causing a major setback in the management of typhoid [2]. Therefore, controlling infections through a nonantibiotic approach is urgently needed. The potential use of *Lactobacillus *to control typhoid fever represents a promising approach, as it may exert protective actions through various mechanisms. Lactobacilli have a long history of safe use, especially in the dairy industry [3]. They resemble a major part of the commensal human mucosal flora [4–8]. Different *Lactobacillus* strains can function as microbial barriers against gastrointestinal pathogens through competitive exclusion of pathogen binding, modulation of the host's immune system, and production of inhibitory compounds, such as organic acid (e.g., lactic acid and acetic acid), oxygen catabolites (e.g., hydrogen peroxide), proteinaceous compounds (e.g., bacteriocins), fat and amino acid metabolites, and other compounds (e.g., reuterin) [9–12]. Several *in vitro* and *in vivo* experimental studies as well as clinical trials have demonstrated the protective role of *Lactobacillus* strains in counteracting a wide range of intestinal infections, such as antibiotic-associated diarrhea, *Helicobacter pylori* gastroenteritis, and urogenital infections [11, 13–15]. However, nearly nothing is known about the antagonistic activity of Lactobacilli against typhoid infection.

The aim of this study was to evaluate the antagonistic activity of some *Lactobacillus* isolates against *Salmonella typhi* by applying the established *in vitro* tests. The results of this study revealed that twelve new potential *Lactobacillus plantarum* candidates satisfy the criteria for *in vivo* and application studies as biotherapeutic agents for controlling typhoid fever.

## 2. Materials and Methods

### 2.1. Microorganisms and Growth Conditions

A total of 32 *Lactobacillus* isolates, recovered and selected as probiotic candidates in a previous study [16], were cultured in MRS broth (Difco) and incubated at 37°C under anaerobic conditions (anaerobic jar supplied with gas generating kits). Eight *Salmonella *isolates were recovered from stool specimens from patients (El Demerdash Hospital and Naser Institute Hospital, both in Cairo, Egypt) having typhoid fever (serologically diagnosed as Widal positive) and included three *Salmonella typhi* (SS6, SS7, and SS8), one *Salmonella paratyphi *A (SS1), and four *Salmonella paratyphi *B (SS2, SS3, SS4, and SS5) isolates. *Salmonella* isolates were grown in BHI broth (Oxoid) at 37°C, unless otherwise indicated. All isolates used in the present study were maintained in 20% glycerol stock at −20°C and subcultured twice prior to performing the experiments.

### 2.2. Cell Line and Growth Conditions

The cell line used in this study was *Vero* cell line (ATCC no. CCL-81), which are kidney epithelial cells derived from the African green monkey, and was purchased from VACSERA, Cairo, Egypt. This cell line was maintained in DMEM (Dulbecco's Modified Eagle Medium; Sigma) supplied with 5% fetal bovine serum (FBS, Sigma). All experiments were performed using *Vero* cells grown (confluent monolayer) in DMEM without FBS in 96-well, flat bottom, tissue culture plates. 

### 2.3. Screening *Salmonella* Isolates for Some Virulence Determinants

The recovered *Salmonella* isolates were screened for some virulence determinants, which included adherence capabilities to, invasion into, and cytotoxicity against mammalian cells.

#### 2.3.1. Adherence and Invasion Assay

This was carried out as described by Plotkowski et al. [17]. The medium submerging the mammalian cell monolayer in the tissue culture plate was first discarded. Aliquots of 200 *μ*L of bacterial suspension were then added to the wells of the tissue culture plate and the plate was incubated for 2 h; then, the monolayer with adherent bacteria was washed 3 times with DMEM-phosphate buffered saline (PBS, pH 7.4), 1 : 1. Quantitative determination of the adherent viable bacteria was carried out depending on the difference between the total number of the bacterial cells (adherent to and uptaken by mammalian cells) and the number of uptaken bacterial cells. The total bacterial number was determined as follows: after washing of the monolayer with DMEM-PBS (1 : 1) medium, lysis of mammalian cells was carried out by treating with 125 *μ*L of lysis solution (0.05% trypsin-EDTA) for 30 minutes at 37°C. Aliquots of the cell lysates were appropriately diluted and plated onto S.S agar plates (*Salmonella-Shigella* agar) for the test isolate. The plates were incubated aerobically for 24 h at 37°C for determination of viable bacterial count. Bacterial invasion was measured by counting only bacteria located into the *Vero* cells [18]. The number of uptaken bacterial cells was determined as follows: after infecting the *Vero* cells with 200 *μ*L (10^8^ CFU/mL) of the test clinical isolate for 3 h and washing of the monolayer with DMEM-PBS (1 : 1), aliquots of 250 *μ*L of gentamicin solution (200 *μ*g/mL) in DMEM medium were added to wells, and the plate was left at room temperature for one hour to kill the adherent bacteria. After removal of gentamicin solution, the mammalian cells were washed three times with DMEM-PBS (1 : 1), treated with the lysis solution, and the number of uptaken cells was then determined as described above for adherence assay.

#### 2.3.2. Determination of Cytotoxicity Using Trypan Blue Assay

Cytotoxicity was assessed in a semiquantitative manner by trypan blue dye exclusion assays [19] as follows: an 18-hour BHI culture of tested clinical isolate was centrifuged, washed twice with PBS, and then resuspended and standardized to 5 × 10^8^ CFU/mL using its culture supernatant. *Vero* cells were grown to a confluent monolayer in 96-well, flat bottom, tissue culture plates. After the cell layer was washed with DMEM, 40 *μ*L (5 × 10^8^ CFU/mL) of test isolate suspension in its culture supernatant and 160 *μ*L DMEM were added to each well, and the control wells contained 40 *μ*L BHI and 160 *μ*L DMEM. After 2, 3, and 5 h of incubation at 37°C, cell culture medium was aspirated off and the wells were washed two times with warm (37°C) DMEM-PBS (1 : 1), and then about 10 *μ*L of 0.4% trypan blue was added to each well and left for 15 min; then the overlaid dye was aspirated. The number and percentage of cells that took up the stain were estimated, with the aid of inverted microscopy. A cytotoxicity score was based on the percentage of stained cells to the total number of cells per field; the average value for five examined fields was determined.

### 2.4. Determination of the Antagonistic Effect of Tested *Lactobacillus* Isolates against a Selected *Salmonella* *typhi* Isolate

#### 2.4.1. Antimicrobial Activity

The radial diffusion assay was used to determine the antimicrobial activity of the cell free culture supernatant (CFCS) of *Lactobacillus* isolates. *Lactobacillus* isolates were grown in MRS broth for 48 h at 37°C. A cell free solution was obtained by centrifuging the culture at 5000 rpm for 15 min, followed by filtration of the supernatant through a 0.2 *μ*m pore-size cellulose acetate filter [20]. *Salmonella typhi *was grown for 18 h at 37°C in BHI. The bacteria were pelleted by centrifugation at 5000 rpm for 15 min, washed once with and resuspended in PBS. A volume containing 10^6^ CFU/mL was added to 20 mL of autoclaved, warm (42°C) Mueller-Hinton agar. After rapid dispersion with a laboratory vortex mixer, the agar was poured into a 20 cm diameter Petri dish to form a uniform layer of approximately 2 mm depth. An 8 mm diameter gel punch was used to make twelve, evenly spaced wells per dish. An aliquot (150 *μ*L) of CFCS of *Lactobacillus* isolate was added to each well and MRS medium was used as a control. After incubation for 18 to 24 h at 37°C, the diameter of the clear zone surrounding each well was measured [21].

#### 2.4.2. Characterization of Antimicrobial Activity

To test the sensitivity to protease, the CFCS was incubated at 37°C for 1 h with and without trypsin (200 mg/mL). To determine if the produced organic acids (lactic acid and acetic acid) in the culture supernatant participate in the CFCS antimicrobial activity, the acidity of CFCS was neutralized using 0.1 N NaOH to pH 7. The remaining activity against pathogenic isolates in both treated samples was determined by the radial diffusion assay [21].

#### 2.4.3. Interference with Adherence and Invasion of a Selected *Salmonella* *typhi* Isolate

*Adherence Inhibition Assay*.* Vero* cell confluent monolayer in the tissue culture plate were washed twice with PBS and then 100 *μ*L (2 × 10^8^ CFU/mL) aliquot, each of *Lactobacillus* and *Salmonella typhi* test isolates suspended in DMEM were added to each well simultaneously, and then the plate was incubated for 2 h at 37°C. The cells were then washed three times with PBS, lysed with 0.05% trypsin-EDTA solution, and the procedure was completed as in the adherence assay. Control wells were treated similarly except that 100 *μ*L DMEM were included instead of *Lactobacillus* suspension. *Salmonella*-*Shigella* (S.S) and MRS agar plates were used as culture media for a viable count of *Salmonella* and *Lactobacillus* cells, respectively.

*Inhibition of Invasion*. The procedure was carried out as described above for adherence inhibition assay, except that wells of both test and control were treated with gentamicin solution before monolayer lysis to determine only the number of invaded cells of the selected *Salmonella typhi* isolate in the presence and absence of tested *Lactobacillus* isolate.

#### 2.4.4. Interference with *Salmonella* *typhi* Growth in CoCulture

The interference of a *Lactobacillus* test isolate with the growth of the selected *Salmonella typhi* isolate was evaluated by incubating a coculture of both isolates and comparing the recovered cells with those obtained from pure cultures of both isolates. For this experiment, a tube containing 10 mL of coculture growth medium (equal quantities of double strength of MRS and Mueller-Hinton broths) was inoculated with 10^5^ CFU/mL each of *Lactobacillus* and *Salmonella *test isolates [22]. The inoculated tubes were incubated at 37°C. After 12 h, the medium was refreshed to limit changes in growth due to pH variation or nutrient consumption; to achieve this, cultures were centrifuged for 15 min at 5000 rpm, and then pellets were resuspended in the same volume initially applied from coculture growth medium. After 24 h total incubation period, bacterial cells were collected by centrifugation (15 min at 5000 rpm) and resuspended in phosphate buffered saline by vortex mixing for 1 min to disrupt all aggregates. Several 10-fold successive dilutions were plated on MRS agar to evaluate the *Lactobacillus* growth and on S.S agar to evaluate the growth of *Salmonella*. The MRS agar plates were incubated for 48 h at 37°C under anaerobic conditions, while S.S agar plates were incubated for 24 h at 37°C.

#### 2.4.5. Inhibition of Cytotoxicity

To assess the cytoprotective effect of tested *Lactobacillus* isolates, confluent monolayer of *Vero* cells in 96-well, flat bottom, tissue culture plates was infected with the tested clinical isolate for 3 h as described previously, after being pretreated with the tested *Lactobacillus *isolate (10^7^ CFU/well) for 1 h. The monolayer was then washed twice with PBS, and the procedure was completed as mentioned in cytotoxicity assay using trypan blue. Two controls were similarly conducted in parallel, the first for clinical isolate using *Vero* cells monolayer untreated with *Lactobacillus*, while the second for *Vero* cells monolayer pretreated with *Lactobacillus* without postinfection with the clinical isolate. The cytoprotective effect was determined depending upon the reduction in the number of stained *Vero* cells that was infected after being treated with tested *Lactobacillus *isolate in comparison to control (infected *Vero* cells without pretreatment with tested *Lactobacillus *isolate).

### 2.5. Susceptibility of Tested *Lactobacillus* Isolates to Some Antimicrobial Agents

This was carried out by determining the minimum inhibitory concentration (MIC) of some antimicrobial agents against tested *Lactobacillus* isolates using microdilution technique described by Henry [23]. The antimicrobial agent stock solution was prepared by dissolving the test agent in the appropriate solvent (water for ciprofloxacin, ethanol for chloramphenicol) [24]. Then, the stock solution of the test agent was diluted in MRS broth to obtain an appropriate concentration range (256 to 1 *μ*g/mL) for each tested agent. The inoculum of the tested *Lactobacillus* isolate was prepared by suspending colonies from MRS agar plates, incubated for 24 h at 37°C anaerobically, in sterile 5 mL 0.85% NaCl solution to a turbidity of McFarland standard 0.5. The suspension was diluted 1 : 100 in MRS broth to be used for inoculation purposes. Wells of the 96-well microtiter plate containing 100 *μ*L aliquots of twofold serial dilutions of the tested agents were inoculated with equal aliquots of the bacterial suspension. The plates were incubated under anaerobic conditions at 37°C for 48 h. Subsequently, MICs were read as the lowest concentration of the antimicrobial agent at which visible growth was inhibited [25].

### 2.6. Determination of Oxygen Tolerance of Tested *Lactobacillus* Isolates

Oxygen tolerance of the tested *Lactobacillus* isolates was determined by comparing their growth under aerobic and anaerobic conditions, according to the method stated by Talwalkar et al. [26]. Stationary phase growth, established from fresh subculture of the tested isolate, was used for inoculation of 1% (v/v) of 10 mL MRS broth contained in 15 mL screw caped test-tube, and 50 mL of the same medium contained in 250 mL conical flask. The screw caped test-tube was incubated under anaerobic conditions (anaerobic jar supplied with gas generating kits), while the conical flask was continuously shaken at 150 rpm on an orbital shaker. Both cultures were incubated at 37°C for 24 h. An aliquot of 100 *μ*L from each culture was taken at different time intervals 0, 6, 12, 18, and 24 h, diluted and plated onto MRS agar plates, and incubated anaerobically for 48 h at 37°C for determination of viable bacterial count. 

### 2.7. Identification of the Selected *Lactobacillus* Isolates That Have Probiotic Potential

Complete identification to the species level was carried using API CHL 50 system (Biomerieux, Marcy l' Etoile, France), a standardized system consisting of 50 biochemical tests for the study of carbohydrate metabolism by microorganisms, and the procedures were conducted according to the manufacturer's instructions.

## 3. Results

### 3.1. Criteria Used for Selection of Clinical Isolates

**Figure 1:**
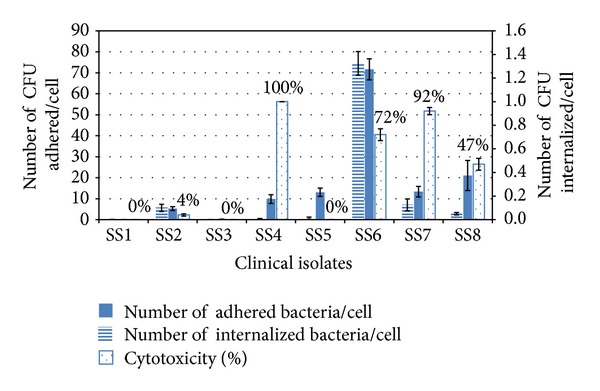
Adherence capacities, invasion capabilities, and cytotoxicity of the tested clinical isolates to *Vero* cells.

Eight *Salmonella* isolates (codes SS1 to SS8) were evaluated for some virulence characters, which included adherence to and invasion into mammalian cells, in addition to their cytotoxic effect. The adherence capacities were expressed as number of adherent bacteria per one *Vero* cell. The results for adherence, invasion, and cytotoxicity are presented in Figure 1 and revealed that *Salmonella* isolate SS6 exhibited the highest adherence to and invasion into *Vero* cells, and a degree of cytotoxicity reached 72%.

The cytotoxicity of washed bacterial cells of tested *Salmonella* isolates in absence of their growth supernatant was undetectable at 2 and 3 h contact time and very low after 5 h (data not shown) while the bacterial cells in their growth supernatant showed different degrees of cytotoxicity with high value for some isolates. The cytotoxicity values after 3 h (Figure 1) were higher than those after 2 h (data not represented), while 5 h contact time caused nearly complete lysis of *Vero* cells for isolates with high cytotoxicity. For examining the cytoprotective effect of tested lactobacilli, *Salmonella* isolate SS6 whth the 3-hour contact time, which gave a pronounced but still submaximal effect was used. As shown in Figure 1, *Salmonella* isolates SS4 and SS7 exhibited cytotoxicity exceeding 90%.

According to the obtained results, *Salmonella *isolate SS6, which exhibited the highest virulence characters (adherence and invasion) and marked cytotoxicity to *Vero* cells, was selected to investigate the antagonistic activities of the *Lactobacillus *isolates. 

### 3.2. Antagonistic Activity of Tested *Lactobacilli* against *Salmonella* *typhi* Isolate SS6

#### 3.2.1. Antimicrobial Activity

The cell free culture supernatants (CFCSs) of 48 h cultures of *Lactobacillus* isolates (32 isolates) were examined for their antimicrobial activity against *Salmonella typhi* isolate SS6 by agar diffusion method; the antimicrobial activity was recorded as the growth free inhibition zone around the wells. Different tested *Lactobacillus* isolates showed variable antimicrobial activities (Table 1). 

#### 3.2.2. Characterization of Antimicrobial Activity

The CFCSs of the 13 *Lactobacillus* isolates (having strong antimicrobial activities against *Salmonella typhi *isolate SS6) were treated to distinguish whether the killing activity was due to the production of acid and/or proteinaceous material such as bacteriocin. The antimicrobial activities of the 13 tested *Lactobacillus* isolates against *Salmonella typhi *isolate SS6 were completely diminished by neutralization with NaOH. When the CFCSs of the tested isolates were treated with trypsin (200 *μ*g/mL), the antimicrobial activities of only four isolates (B2a, B2b, B10, and L4) decreased, while the other tested isolates retained their antimicrobial activities.

#### 3.2.3. Interference with *Salmonella* *typhi* Adherence and Invasion

The ability of *Lactobacillus* isolates to prevent *Salmonella typhi* adherence and invasion was examined through incubating a mixed suspension of tested *Lactobacillus* isolate and *Salmonella typhi* isolate SS6 with *Vero* cells for two hours. The obtained results showed that the adherence to and invasion into *Vero* cells by *Salmonella typhi* in presence of lactobacilli varied greatly, as shown in Tables 2 and 3. 

From the previous results, twelve *Lactobacillus *isolates C4, C7, C8, B2a, B10, B11, L4, L36, L37, L38, L39, and L47 showed strong antimicrobial activities as well as high interference with *Salmonella typhi* isolate SS6 invasion into *Vero* cells. These isolates were selected to be examined for other antagonistic activities against *Salmonella typhi*. The characters of these isolates are summarized in Table 4.

#### 3.2.4. Interference with *Salmonella* *typhi* Growth in Coculture

**Figure 2:**
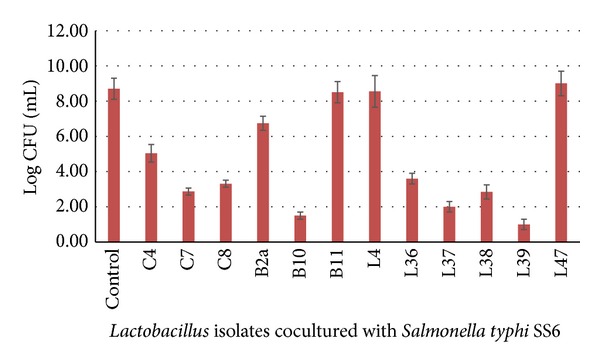
Growth of *Salmonella typhi* (SS6) when cocultured with some selected *Lactobacillus* isolates after 24 h.

The capability of the selected *Lactobacillus* isolates to inhibit the *in vitro* growth of *Salmonella typhi* was evaluated in a coculture experiment. The results represented in Figure 2 showed that nine *Lactobacillus* isolates inhibited the growth of *Salmonella typhi *isolate SS6 dramatically after 24 h of incubation, while three *Lactobacillus* isolates (B11, L4, and L47) nearly did not affect the growth of the test isolate. However, the growth of tested *Lactobacillus* isolates was not affected by the simultaneous presence of *Salmonella typhi isolate SS6 *(data not shown).

#### 3.2.5. Protective Effect of Tested *Lactobacilli* against *Salmonella* *typhi* Cytotoxicity

**Figure 3:**
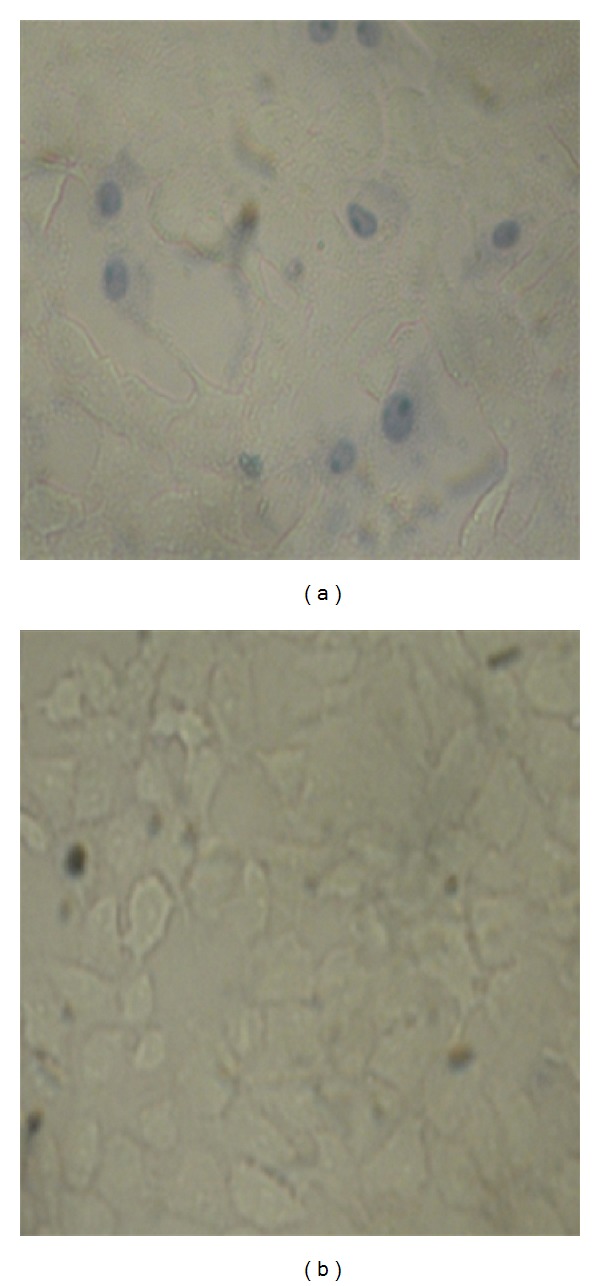
Cytotoxic effect of *Salmonella typhi* isolate (SS6) on untreated and *Lactobacillus* treated *Vero* cells. (a) *Vero* cells infected with *Salmonella typhi* isolate SS6 for 3 h, and (b) *Vero* cells infected with *Salmonella typhi* isolate SS6 for 3 h after their treatment with *Lactobacillus* isolate C8 for 1 h.

**Figure 4:**
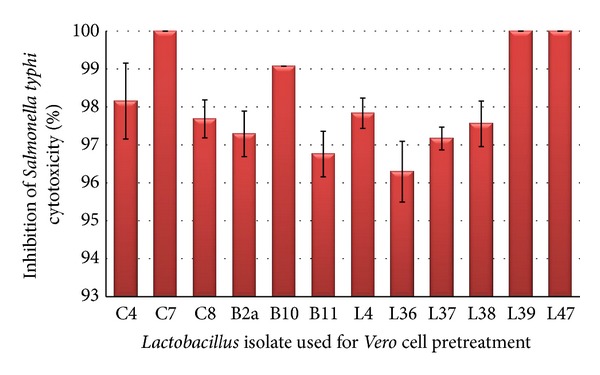
Effect of pretreatment of *Vero* cells with different *Lactobacillus* isolates on cytotoxicity of *Salmonella typhi* isolate SS6.

The cytoprotective effect of tested *Lactobacillus* isolates (12 isolates) against *Salmonella typhi* isolate SS6 cytotoxicity was evaluated by measuring inhibition in cytotoxicity due to the presence of lactobacilli. The tested *Lactobacillus* isolates were examined firstly to test if they have any cytotoxic potential. The results showed that the tested isolates had not any cytotoxic potential (data not shown). The cytoprotective effect on *Vero* cells pretreated with tested *Lactobacillus* isolates followed by infection with *Salmonella typhi* was assessed by trypan blue exclusion assay. As a representative example, Figure 3 showed that after staining *Vero* cells with trypan blue, cells infected with *Salmonella typhi* isolate SS6 showed a high degree of cytotoxicity (lysed cells plus other cells stained blue) while *Vero* cells pretreated with *Lactobacillus *isolate C8 and infected with *Salmonella typhi isolate SS6* showed high viability (no cell lysis and absence of blue stained cells). In terms of quantity, the protective effect of the tested* Lactobacillus *isolates against *Salmonella typhi *isolate SS6 cytotoxicity on *Vero* cells is represented in Figure 4. The results showed that all tested *Lactobacillus* isolates nearly caused complete inhibition of *Salmonella typhi* cytotoxicity on *Vero* cells. 

### 3.3. Susceptibility of Tested *Lactobacillus* Isolates to Some Antimicrobial Agents

This was carried out by determining the MIC of certain antimicrobial agents against the tested *Lactobacillus* isolates. Two antimicrobial agents, ciprofloxacin and chloramphenicol (the drugs of choice for typhoid fever treatment), were tested against the twelve *Lactobacillus* isolates that have promising antagonistic activities against *Salmonella typhi* isolate SS6 (C4, C7, C8, B10, B11, L4, L36, L37, L38, L39, L47, and B2a). The results (Table 5) revealed that MIC value of ciprofloxacin was much lower for *Salmonella typhi* isolate SS6 as compared to lactobacilli, while the MIC value of chloramphenicol for that isolate was nearly comparable to those for *Lactobacillus* isolates. 

### 3.4. Oxygen Tolerance of Tested *Lactobacillus* Isolates

The oxygen tolerance of *Lactobacillus* isolates was evaluated by comparing their growth under aerobic and anaerobic conditions. The growth of the 12 *Lactobacillus* isolates was examined at different time intervals (0, 6, 12, 18, and 24 h) at 37°C under aerobic and anaerobic conditions. The obtained results showed that all tested *Lactobacillus* isolates were able to grow under aerobic conditions, as shown in Table 6.

### 3.5. Identification of Tested *Lactobacillus* Isolates Having Promising Antagonistic Activities against *Salmonella* *typhi*

The results revealed that the tested isolates belong to *Lactobacillus plantarum* with confidence percentage equal to 99.9%.

## 4. Discussion

In the present study we evaluate the virulence of eight *Salmonella *isolates: the most virulent isolate, *Salmonella typhi* SS6 was selected (it showed high invasion capability as well as dramatic cytotoxicity to *Vero* cells) for examining the antagonistic activity of *Lactobacillus *isolates. This antagonistic activity included; secretion of antimicrobial compounds, interference with adherence, and invasion of *Salmonella typhi* isolate SS6 into epithelial cells, in addition to interference with its growth and cytotoxicity. The antimicrobial activity of the cell free culture supernatant of the 32 *Lactobacillus *isolates against *Salmonella typhi* isolate SS6 was evaluated. The obtained results (Table 1) revealed that 13 isolates showed relative strong activity (inhibition zone ≥ 15 mm) and seven isolates showed moderate activity (inhibition zone < 15–10 mm). Several studies reported that lactobacilli produce a wide range of antibacterial compounds, including sugar catabolites such as organic acids (e.g., lactic acid and acetic acid); oxygen catabolites such as hydrogen peroxide; and proteinaceous compounds such as bacteriocins [10–12, 27, 28].

The CFCSs of the 13 *Lactobacillus* isolates (B2b, C8, B10, B11, L4, L37, L36, L38, C4, L47, C7, B2a, and L39) having strong antimicrobial activities against *Salmonella typhi *isolate (SS6) exerted their antimicrobial activities only in acidic pH, and the activity diminished completely at pH 7. In agreement with these results, several studies reported a lack of inhibitory activity of pH adjusted culture supernatant [22, 29]. Lin et al. [30] reported that when *L. acidophilus* LAP5 strain was cultured in MRS broth for 20 h, the pH of the culture supernatant was found to decrease to 3.78. As these cultured broths were neutralized to pH 7.2, the inhibitory activity to pathogenic bacteria became negligible. The antimicrobial activities of the CFCS of *Lactobacillus* isolates B2b, B2a, L4, and B10 decreased after treatment with trypsin, while those of other tested *Lactobacillus* isolates retained their activities. According to the obtained results, the antimicrobial activities of the most tested *Lactobacillus* isolates were attributed to acid, while the antimicrobial activities of *Lactobacillus* isolates B2b, B2a, L4, and B10 could be attributed to proteinaceous material, which is only active at acidic pH. Many authors have associated high antagonistic activity of lactobacilli with production of organic acids resulting in pH decrease [29, 31, 32]. Hütt et al. [29] revealed a correlation between the pH decreases, amount of lactic acid produced, and rank of antimicrobial activity of probiotic strains. De-Keersmaecker et al. [33] reported also that the antimicrobial activity of *Lactobacillus rhamnosus* against *Salmonella typhimurium *was due to accumulation of lactic acid. Fayol-Messaoudi et al. [34] observed that the complete inhibition of *S. typhimurium* SL1344 growth results from a pH-lowering effect. In addition, Cook and Sellin [35] reported that organic acids not only fulfill a barrier effect on pathogenic bacteria, but also play a crucial role in the maintenance of the health of the colon. Millette et al. [36] found that the bactericidal effect of *Lactobacillus* strains was characterized as the production of organic acids, in combination with the production of a bacteriocin-like protein which is active in acidic condition. It has also been reported that *Lactobacillus *sp. strain GG, isolated from the feces of a normal person, produced a substance with potent inhibitory activity in the pH range between 3 and 5 against a wide variety of bacterial species including Gram positive and Gram negative [37]. Rammelsberg and Radler [38] showed that the bacteriocin from *Lactobacillus brevis* or *L. casei* loses its activity at neutral pH value.

The first step in *Salmonella* pathogenesis is the adhesion/invasion to specific intestinal epithelial cells. This event is a prerequisite for the subsequent steps in pathogenesis that lead to mucosal infection, systemic spread, and disease [30]. It has been widely reported that adhesion of *Lactobacillus* strains to mucosa eliminates pathogen adhesion, in this way reducing colonization and prevents infection [39]. In the present study, the tested 32 *Lactobacillus* isolates were investigated for possible blockage of *Salmonella typhi *isolate SS6 adherence to *Vero* cells. Nine *Lactobacillus* isolates inhibited *Salmonella typhi *adherence by more than 50% and 18 isolates inhibited adherence by less than 50% (Table 2). In contrary, increased measured values of *Salmonella typhi *adherence relative to control were observed in presence of 5 *Lactobacillus* isolates (C9, L61, L62, L63, and B2a), and this may be due to coaggregation of *Lactobacilli* with *Salmonella typhi *test isolate. The *in vitro* inhibition of Gram negative pathogens adhesion to eukaryotic cell lines has been reported for several probiotic strains, such as *L. johnsonii* La1*, Bifidobacterium* CA1 andF9, and *L. acidophilus LB* [40–43]. Maragkoudakis et al. [44] also reported reduction of the adhesion of *E. coli* CFA1 and *S. typhimurium* SL1344 to Caco-2 cells, when the Caco-2 cells were previously challenged with strains *L. plantarum* ACA-DC 146 and *L. paracasei* subsp. *paracasei* ACA-DC 221.

Inhibition of the invasion of *Salmonella* into epithelial cells is the first step in disease prevention, as it is critical to initiate the infection [45]. In the present study, 32 *Lactobacillus* isolates were evaluated for their interference with the *Salmonella typhi *isolate SS6 invasiveness into *Vero* cells. According to the obtained results, the invasion capability of *Salmonella typhi *isolate SS6 in presence of *Lactobacillus* varied greatly (Table 3). Twenty-five *Lactobacillus* isolates were able to inhibit *Salmonella typhi *invasion by more than 50%, and 7 isolates inhibited *Salmonella typhi *invasion into *Vero cells* by less than 50%. The results showed that six *Lactobacillus* isolates inhibited *Salmonella typhi *isolate SS6 invasion into *Vero cells *by more than 90%. Several studies reported that the adhering human *Lactobacillus *strains inhibited association and invasion of host cells by several enterovirulent bacteria. Coconnier and coworkers [41, 46] reported that both living and heat-killed *Lactobacillus* strains were able to protect intestinal cells against attachment and invasion of a large variety of enterotoxigenic and enteroinvasive bacteria. Makras et al. [47] found that lactic acid produced by lactobacilli was responsible for significant inhibitory effects upon invasion of *Salmonella *into Caco-2/TC7 cells. The possible mechanism of competitive exclusion of *Salmonella typhi* by *Lactobacillus* isolates seems to be a result of a nonspecific steric hindrance or a specific blockage of receptors sites. It was observed that the *Lactobacillus* isolates, which resulted in increased measured values of *Salmonella typhi *adherence relative to control (Table 2), were able to block *Salmonella typhi *invasion into *Vero* cells by more than 50%. A coaggregation between these *Lactobacillus* isolates and *Salmonella typhi *isolate SS6 could be suggestive for prevention of *Salmonella typhi *internalization into *Vero* cells in spite of showing high adherence values. In accordance to our finding, Golowczyc et al. [18] found that coincubation of *Salmonella* with coaggregating *Lactobacillus* strains significantly decreased its capacity to invade Caco-2/TC-7 cells. 

Taken together, in the present study twelve *Lactobacillus *isolates (C4, C7, C8, B2a, B10, B11, L4, L36, L37, L38, L39, and L4) showed strong antimicrobial activity as well as high interference with invasion of *Salmonella typhi *isolate SS6 into *Vero* cells. The possible interference of these selected lactobacilli with the growth of *Salmonella typhi *isolate SS6 was investigated in coculture experiment, since a correct assessment of interaction between a probiotic and pathogen can be obtained when they are cultured in the same medium and share the same environmental growth conditions. Different culture media were evaluated with the aim of finding medium able to support the growth of both the enteropathogenic *Salmonella typhi *isolate SS6 and *Lactobacillus* isolates. The obtained results revealed that most *Lactobacillus* isolates dramatically inhibited the growth of *Salmonella typhi *to undetectable levels, while the growth of lactobacilli was not influenced by the presence of *Salmonella typhi *(Figure 2). Seven *Lactobacillus *isolates not only showed interference with *Salmonella typhi* growth, but also showed strong killing activity. This interference with *Salmonella typhi* growth may be attributed to decreased pH levels, competition for substrates, and the production of substances with a bactericidal or bacteriostatic action, including bacteriocins [48]. Another possible mechanism is the coaggregation between *Lactobacillus *isolate and *Salmonella typhi* isolate. Mastromarino et al. [49] reported that such coaggregation provides large contact areas around the pathogen with consecutive rise of inhibiting substances in this microenvironment produced by lactobacilli. In agreement with our findings, Fayol-Messaoudi et al. [34] investigated the antibacterial activity of *Lactobacillus plantarum* strain ACA-DC287 isolated from a Greek cheese and determined that the coculture of this strain with *S. typhimurium* resulted in the killing of the pathogen, due to nonlactic acid molecules. 

Major factors contributing to *Salmonella* pathogenesis are its ability to invade epithelial cells and causing of cellular damage. In the present study, the *Salmonella typhi* isolate SS6 showed marked cytotoxicity to *Vero *cells. The possible protective role of lactobacilli was investigated. Our findings indicate that the tested *Lactobacillus* isolates did not adversely affect the integrity and viability of epithelial cells. The results revealed that preincubation of the *Vero *cells monolayer with viable lactobacilli reduced the cytotoxicity of *Salmonella typi* isolate SS6 to undetectable levels (Figures 3 and 4). *Lactobacillus *isolates act as a barrier to avoid the direct contact between *Salmonella typhi* and *Vero* cell and prevent its invasion; consequently, they protect *Vero* cells from the damage encountered by this pathogen. In accordance with our finding, it was reported that *L. rhamnosus GG* reduce the adhesion and cytotoxicity of *Salmonella enterica* serovar *typhimurium* [50]. 

The selected *Lactobacillus* isolates (C4, C7, C8, B10, B11, L4, L36, L37, L38, L39, L47, and B2a) that showed high probiotic potential against the enteropathogenic *Salmonella typhi* isolate SS6 are considered as probiotic candidates (Table 4). These probiotic candidates were further subjected to some tests that may affect their use, such as susceptibilities to antimicrobial agents that are commonly used in treatment or their proliferation and production, such as oxygen tolerance. Evaluating the susceptibility of probiotic candidates to antimicrobial agents has great clinical importance, since it enables the concomitant use of probiotic with appropriate doses of antimicrobial agents to treat typhoid fever. Two antimicrobial agents ciprofloxacin and chloramphenicol (the drugs of choice for typhoid fever treatment) were tested against the twelve *Lactobacillus* isolates that were active against *Salmonella typhi* isolate SS6. The results (Table 5) revealed that MIC value of ciprofloxacin was much lower for *Salmonella typhi* isolate SS6 as compared to those of tested lactobacilli, while the MIC value of chloramphenicol for that isolate was nearly comparable to those of tested lactobacilli. Consequently, ciprofloxacin could be also used in combination with the tested *Lactobacillus* isolates to treat typhoid fever.

In order to exert their functional properties, probiotics need to be delivered to the desired sites in an active and viable form. The viability and activity of probiotics in the products have been frequently cited as a prerequisite for achieving numerous beneficial health benefits. Therefore, these bacteria must survive during processing, in the preparation during shelf life and during transit through the gastrointestinal tract [51]. Consequently, the selection of probiotic strains is based not only on the functional criteria but also on additional technological aspects. Among the reasons responsible for the loss in probiotic viability, cell death due to oxygen toxicity is considered a significant factor [52–54]. Oxygen can affect the probiotic culture during processing and it can also enter the product through packaging materials during storage. Strains of *Lactobacillus* and *Bifidobacterium* spp. are microaerophilic and anaerobic, respectively. They lack an electron-transport chain, which results in the incomplete reduction of oxygen to hydrogen peroxide. Furthermore, they are devoid of catalase, thus incapable of converting hydrogen peroxide into water. This results in the intracellular accumulation of hydrogen peroxide and consequently death of the cell [54]. In the present study, the oxygen tolerance of *Lactobacillus* isolates was evaluated by comparing their growth under aerobic and anaerobic conditions. The growth of the 12 probiotic *Lactobacillus* candidates was examined at different time intervals; 0, 6, 12, 18, and 24 h at 37°C under aerobic (shaking at 150 rpm) and anaerobic conditions. The results (Table 6) revealed that all tested *Lactobacillus* isolates were able to grow well under aerobic conditions; however, some isolates showed lower growth patterns in aerobic conditions. Their ability to grow in aerobic conditions suggested that these isolates possessed a mechanism to overcome the deleterious effects of oxygen toxicity. Archibald and Fridovich [55] reported that *L. plantarum* has a capacity for scavenging O_2_, which is comparable to that observed in aerobically grown *Escherichia coli. L*. *plantarum* demonstrated that its high intracellular level of Mn (II) takes the place of superoxide dismutase in scavenging O_2_. They also reported that *L*. *plantarum* strains are more resistant to lethality of aerobic conditions than *L*. *acidophilus* strains, since they possess high intracellular levels of Mn (II). In the present study, the tested *Lactobacillus* isolates showed oxygen tolerance in addition to their acid and bile tolerance. In agreement with our finding, Kim et al. [56] suggested that bacteria can exhibit a common stress response offering cross protection against a variety of environmental factors. 

The tested *Lactobacillus* isolates with potential probiotic properties showed promising antagonistic activity against *Salmonella typhi *and were fully identified to the species level using API 50 CHL system. The results revealed that all isolates belong to *Lactobacillus plantarum*. This species is a versatile lactic acid bacterium that is encountered in a range of environmental niches including dairy, meat, and many vegetable fermentations. Moreover, it is commonly found in the human gastrointestinal tract (GIT) [57]. Regarding its safety, *L. plantarum* has a long history of natural occurrence and safe use in a variety of food products [57]. 

## 5. Conclusion

The results of the present study revealed that twelve* Lactobacillus plantarum *isolates (C4, C7, C8, B2a, B10, B11, L4, L36, L37, L38, and L39) could protect against *Salmonella typhi* infection through interference with both its growth and its virulence determinants such as adherence, invasion, and cytotoxicity. The concomitant use of these *Lactobacillus plantarum *isolates with ciprofloxacin to manage typhoid fever could be acceptable, since the MIC values of ciprofloxacin were higher with tested lactobacilli as compared to those with *Salmonella typhi. *These probiotic candidates are oxygen tolerant and as a consequence can retain viability during processing and storage. Therefore, they could be novel therapeutic agents for prevention and treatment of typhoid fever after being subjected to *in vivo* and application studies.

## Figures and Tables

**Table 1:** Categorization of the antimicrobial activity of the tested *Lactobacillus* isolates against *Salmonella typhi* isolate (SS6).

Antimicrobial activity	Diameter of inhibition zone (mm)*	Number of isolates	Percentage relative to total number of isolates	Isolates
Strong	≥15	13	40.63%	B2b, C8, B10, B11, L4, L37, L36, L38, C4, L47, C7, B2a, and L39
Moderate	<15–10	7	21.88%	C9, B1, L22, L21, L53, B9, and L61
Weak	≤10	2	6.25%	C10 and LS
No activity	No	9	31.25%	C5, B3, L5′, L24, L33, L49, L50, L62, L63, and S1

*Punch diameter = 8 mm.

**Table 2:** Interference of *Lactobacillus* isolates with adherence of *Salmonella typhi* isolate (SS6) to *Vero* cells.

Degree of interference	Number of isolates	Isolate code	% Inhibition of adherence
≥50% inhibition of adherence	9	B3	91.43
		C7	80
		B10	75
		L5′	72.86
		L22	71.43
		C10	70
		L53	60
		B11	59
		L38	55

<50%–25% inhibition of adherence	9	L21	46.03
		C8	45
		L33	42.86
		LS	42.03
		C4	37
		B1	36.23
		L36	35
		B9	30
		L4	26.64

<25%–5% inhibition of adherence	6	C5	20
		S1	16.3
		L39	15
		L49	13
		L50	10
		L24	9

No inhibition of adherence	3	B2b	3
		L37	2
		L47	0

Altered effect*	5	C9	−30
		L62	−102
		L63	−134
		L61	−137
		B2a	−200

*Increased measured values relative to control which may be due to coaggregated *Salmonella typhi* cells on the *Vero* cells monolayer-pre-adhered *Lactobacillus* cells.

**Table 3:** Interference of *Lactobacillus* isolates with invasion of *Salmonella typhi *isolate SS6 into *Vero* cells.

Degree of interference	Number of isolates	Isolate code	% Inhibition of invasion
≥90% inhibition of invasion	6	C5	98
		L38	97.1
		L22	91.55
		L37	91
		L47	91
		L39	90.9

<90%–80% inhibition of invasion	9	L4	89.78
		L36	85
		B1	84.89
		L53	84.85
		C7	84.4
		L62	84
		L21	83.16
		L63	81.82
		C4	80

<80%–50% Inhibition of invasion	10	C8	78.88
		C9	78.79
		B9	78.79
		L33	74.65
		B10	72.73
		L61	72.73
		B11	69.7
		C10	66.67
		LS	55.56
		B2a	54.55

>50% inhibition of invasion	3	S1	47.1
		L5′	40.74
		L24	33.33

No inhibition	4	B2b	0
		B3	0
		L49	0
		L50	0

**Table 4:** Summary of antimicrobial activity and interference with *Salmonella typhi* (SS6) invasion of twelve selected *Lactobacillus* isolates.

*Lactobacillus* isolate	Antimicrobial activity^a^	% Inhibition of *Salmonella typhi* invasion
C4	15	80
C7	17	84.4
C8	21	78.8
B2a	15	54.5
B10	15	72.7
B11	16	69.7
L4	19	89.7
L36	17	85
L37	20	91
L38	18	97.1
L39	15	90.9
L47	15	91

^a^Antimicrobial activity (expressed as diameter of inhibition zone in mm as determined by agar diffusion method).

**Table 5:** MICs of some antimicrobial agents against probiotic *Lactobacillus* candidates.

Isolate code	MIC (*μ*g/mL)	
	Ciprofloxacin	Chloramphenicol
C4	8	32
C8	16	64
B10	32	16
L4	4	64
L37	16	32
L38	32	32
C7	4	32
L36	64	16
L39	16	32
L47	8	16
B2a	4	32
B11	16	32
*Salmonella typhi* SS6	0.5	32

**Table 6:** Specific growth rates of the tested *Lactobacillus* isolates under aerobic and anaerobic conditions.

*Lactobacillus* isolates	Specific growth rate (h^−1^)		Growth after 24 h (Log CFU/mL)	
	Anaerobic	Aerobic	Anaerobic	Aerobic
B10	0.691	0.537	8.1	7.9
B11	0.384	0.384	7.3	7.3
C4	0.614	0.384	8.5	8.4
C7	0.614	0.461	8.3	8.1
C8	0.614	0.384	8.5	8.2
B2a	0.537	0.307	8.1	7.9
L4	0.384	0.288	8.3	8.3
L36	0.537	0.384	8.4	8.2
L37	0.614	0.614	8.4	8.3
L38	0.691	0.384	8.2	7.6
L39	0.614	0.537	8.3	8.3
L47	0.614	0.230	9	8.7
